# Fish populations surviving estrogen pollution

**DOI:** 10.1186/1741-7007-12-10

**Published:** 2014-02-10

**Authors:** Claus Wedekind

**Affiliations:** 1 Department of Ecology and Evolution, University of Lausanne, Biophore, Lausanne, 1015, Switzerland

## Abstract

Among the most common pollutants that enter the environment after passing municipal wastewater treatment are estrogens, especially the synthetic 17α-ethinylestradiol that is used in oral contraceptives. Estrogens are potent endocrine disruptors at concentrations frequently observed in surface waters. However, new genetic analyses suggest that some fish populations can be self-sustaining even in heavily polluted waters. We now need to understand the basis of this tolerance.

See research article: http://www.biomedcentral.com/1741-7007/12/1

## Commentary

Two key questions in conservation biology are whether and how natural populations are affected by anthropogenic changes of their habitat, and whether and how they can adapt to them [1]. Undoubtedly one of the major challenges to water-dwelling organisms is chemical pollution through residues in effluents of wastewater treatment plants. An important group of such pollutants includes hormones like the natural estrone and 17β-estradiol (E2), and the synthetic 17α-ethinyloestradiol (EE2) that was first synthesized in the 1930s and mimics E2 in many formulations of oral contraceptives. EE2 plays an extraordinary role as a pollutant because of its high estrogenic potency and because it is, in the aquatic environment, more stable and persistent than natural estrogens. EE2 is now commonly found in surface waters at concentrations around 1 ng/L [2], but higher concentrations have frequently been reported or are expected, especially so in densely populated southern England that includes the study area of Hamilton *et al*. [3] and that has ‘… some of the highest proportions of [wastewater treatment plant] effluents in rivers known globally’ [3].

Exposure to estrogens can have various detrimental effects in fish. It can reduce general viability, induce gonadal malformations or feminization of genetic males, or lead to sterilization [4,5]. Concentrations below 1 ng/L can induce vitellogenin production in male zebrafish (*Danio rerio*) and rainbow trout (*Oncorhynchus mykiss*) [6], and higher concentrations of EE2 that are sometimes found in surface waters have been demonstrated to cause sex reversal in the laboratory (for example, 10 ng/L [7]). Indeed, exposure to effluents of wastewater treatment plants has led to all-female populations in field experiments [8]. Sex reversal is possible in many fishes where sex is genetically determined, that is, treatment of fish with hormones can functionally override the genetic sex. Estrogens or androgens are therefore widely used in aquaculture to manipulate gender (for example, if one sex is preferred for economic reasons), but as pollutants they can be serious threats to natural populations (see below). It is therefore important to study the fate of fish populations that live in heavily polluted rivers to learn more about the damaging effects of estrogens, especially when migration barriers leave fish populations essentially two options: cope with the problem or perish.

**Figure 1.**
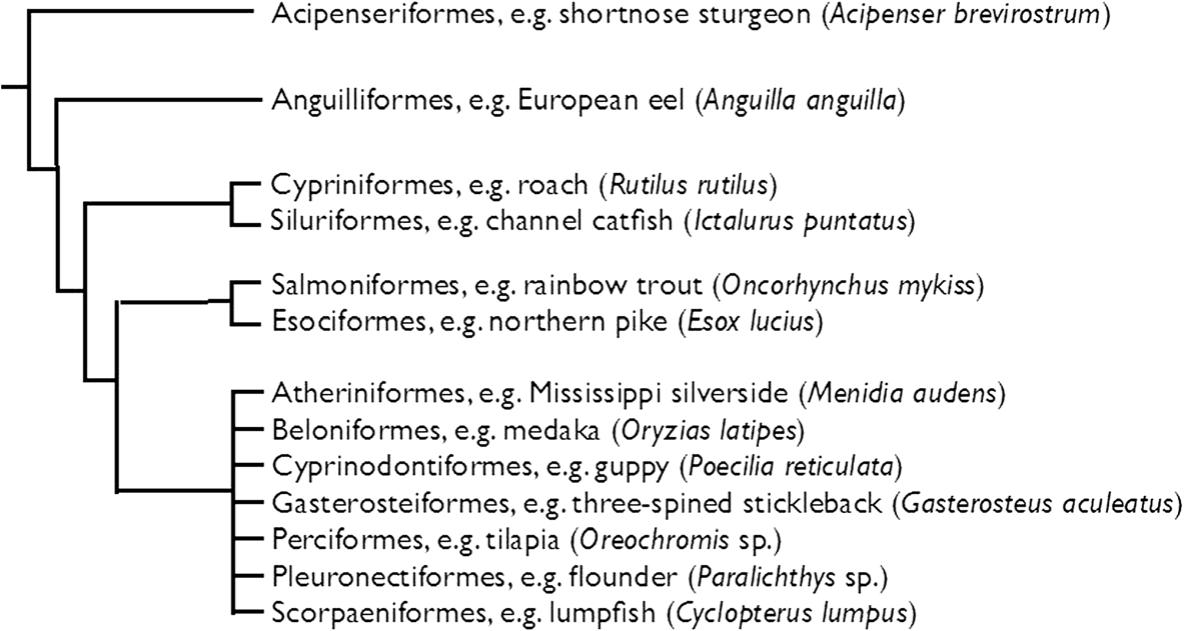
**Major orders of ray-finned fish in which exposure to hormones or hormone-active substances have been shown to cause sex reversal or severe aberrations in gonadal development.** See references in [4,5].

Hamilton *et al*. [3] concentrated on the common roach (*Rutilus rutilus* L.), a cyprinid fish that typically matures at the age of two or three years, feeds mainly on plant material and invertebrates, can stand temperatures of up to about 30°C, and is believed to be more tolerant to organic pollutants than most other fish. This may explain why roach have for a long time been very common in southern England where pollution and other anthropogenic insults to aquatic habitats have a comparatively long history. Hamilton and colleagues’ [3] field study builds on experimental and modeling work that has been done earlier on roach (from this area), on other cyprinids, and on other fin-rayed fish (Figure 1) and that demonstrates a high sensitivity of fish to estrogen exposure. The number of laboratory studies on effects of estrogen pollution is impressive and has been complemented by population models (reviewed in [9]) and phylogenetic analyses (for example, [5]).

**Figure 2.**
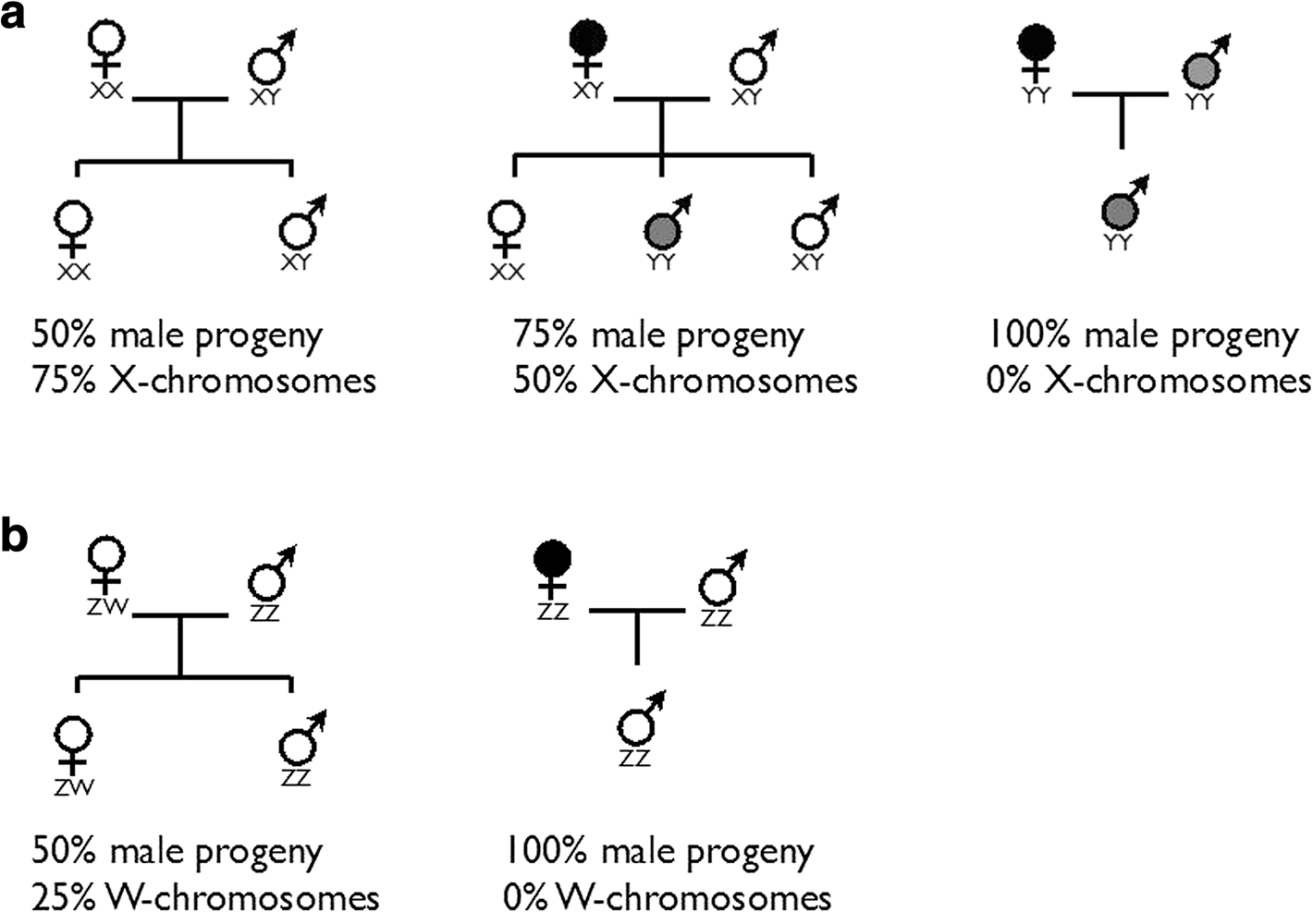
**Mating types, family sex ratios, and corresponding frequencies of Y or W chromosomes in populations affected by estrogen-induced feminization. (a)** XY/XX sex determination system. **(b)** ZZ/ZW sex determination system. Black symbols: phenotype-genotype mismatch caused by feminization. Gray symbols: new karyotypes among the progeny of feminized individuals.

Estrogen pollution threatens wild populations because of its negative effects on the vital rates (survival, growth, and fecundity), family sex ratios (Figure 2), the frequencies of sex chromosomes (Figure 2), and genetic variation and the evolutionary potential of populations. The effects of feminization on population sex ratios may often be counter intuitive: it will first produce female-biased population sex ratios, but because male genotypes are more prevalent among the progeny of feminized individuals (Figure 2), population sex ratios can develop, over time, into anything between heavily female-biased or heavily male-biased, depending on the temporal dynamics of the pollution, the viability and reproductive success of the various genotype-phenotype combinations, and their specific susceptibility to feminization at different developmental stages [10]. We would expect the occurrence of YY individuals in populations with an XY/XX sex determination system (Figure 2; in contrast to mammals, YY individuals are often viable in fish because Y chromosomes are typically far less damaged in fish than they are in mammals [9]). It is even possible that sex chromosomes go extinct (Figure 2), in that populations may lose their genetic sex determination and become dependent on the production of females through estrogen pollution. Ceasing the pollution could then create dramatic negative effects on fish populations.

A population’s evolutionary potential to adapt to new environments depends critically on the amount of genetic variation within the population. Estrogen pollution is predicted to reduce this genetic variation because uneven sex ratios typically lead to increased variance in individual reproductive success (for example, if the sex ratio is female-biased, females are more likely to share mates and the average relatedness in the next generation will be increased). Population censuses can then be misleading. Hamilton *et al*. [3] therefore determined microsatellite genotypes to estimate the genetically effective population size *N*_*e*_ at 28 different sample sites (in total nearly 1,800 individuals were typed for 14 microsatellite loci each). They deduced from pairwise comparisons between sampling locations that the genetic exchange between locations was very low. Low migration allows for comparing *N*_*e*_ estimates with the levels of estrogen pollution at the different sites. Hamilton and colleagues found no evidence for significant effects of estrogen pollution on roach populations. They concluded that despite the strong expectancies about the negative effects of the pollutants, (*i*) there are wild populations of roach that are somehow able to persist in high concentrations of estrogen pollution, and (*ii*) estrogen pollution may not even be among the most important drivers that determine roach breeding population size. However, the authors stress that they could still have missed an estrogen-driven reduction of *N*_*e*_ by up to 65% at the most polluted sites because of statistical noise in their data.

Much has been done to better understand the effects of exogenous estrogens in different fish species and at different developmental stages. Population models based on these insights predict reduced population growth, distorted sex ratios, and extinctions. None of these predictions could be verified in populations of roach that have been exposed to high estrogen concentrations over several generations. Such results cannot tell us much about the tolerance of other species or populations. However, they tell us that we have not yet sufficiently understood the damaging effects of major chemical pollutants to fish at the population level. Key questions for future research are: what is the potential of fish to cope with environmental stress like estrogen pollution, and what is the potential of fish to adapt to these challenges?
